# Comparison of Soft Tissue Chin Thickness in Adult Patients With Various Mandibular Divergence Patterns

**DOI:** 10.7759/cureus.59150

**Published:** 2024-04-27

**Authors:** Muhammad Noman, Gulsana Hashmi, Munawar Manzoor Ali, Usman Yousaf, Mazhar Hussain, Rida Mujeeb

**Affiliations:** 1 Orthodontics and Dentofacial Orthopaedics, Sharif Medical and Dental College, Lahore, PAK; 2 Orthodontics and Dentofacial Orthopaedics, University College of Medicine and Dentistry, University of Lahore, Lahore, PAK; 3 Orthodontics and Dentofacial Orthopaedics, Azra Naheed Dental College, Superior University, Lahore, PAK; 4 Operative Dentistry and Endodontics, Fatima Memorial Hospital College of Medicine and Dentistry, Lahore, PAK

**Keywords:** cephalometric study, orthognathic hypo divergent, hyper divergent, soft tissue chin, mandibular divergence

## Abstract

Objective

This study aimed to compare the soft tissue chin (STC) thickness at different levels in patients presenting for orthodontic treatment with different vertical facial types.

Materials and methods

This comparative cross-sectional study was conducted at Sharif Medical & Dental College, Lahore, Pakistan, on 195 subjects. Patients presenting for orthodontic treatment, both genders, aged from 18 to 32 years, and Pakistani nationals were included. Patients with any craniofacial deformity, syndrome, cleft lip and palate, previous orthodontic or orthognathic treatment, and multiple missing teeth and prostheses in edentulous areas were excluded. Vertical facial patterns and STC thickness were recorded from pre-treatment lateral cephalograms. One-way analysis of variance (ANOVA) was applied to compare STC among various vertical facial patterns. Post-hoc analysis was done using the Tukey test.

Results

There were 126 females (64.62%) and 69 males (35.38%). The mean age was 21.66 ± 3.44 years. All three soft tissue chin thickness distances significantly differed among vertical facial patterns (p<0.001). Multiple comparisons show that the distance between soft and hard tissue pogonion was insignificant between low and normal angle facial heights (p=0.5). Similarly, no significant difference was observed for the distance between soft and hard tissue menton in low and normal angle subjects (p=0.4). The rest of the multiple comparisons were statistically significant (p<0.05).

Conclusion

The STC thickness is significantly associated with vertical facial divergence. While planning orthognathic surgery or genioplasty of the mandible, due consideration should be given to vertical divergence of the face to avoid unwanted facial changes.

## Introduction

Soft tissue chin (STC) thickness and morphology are significant for orthodontists and plastic surgeons while providing patient care [1]. The aim of orthodontic treatment is not limited to dental and hard tissue correction. However, the final aim of this treatment modality is to position teeth and skeletal tissue to achieve a normal and balanced soft tissue profile [2]. Treatment success and failure in orthodontics directly correlate with soft tissue balance [3].

Five main soft tissue entities make the facial profile [4]. These components are the submental-cervical region, chin, lips, nose, and forehead. The interplay among these five components creates an attractive or abnormal profile [5].

The lower face is framed by the nose above and the chin below, which defines the aesthetics. Most of the time, the rhinoplasty is carried out in coordination with chin morphology and vice versa. A case with lip protrusion can usually occur if the chin is prominent. A non-extraction treatment is sometimes justified in patients with good chin morphology [6,7].

Vertical face growth is the last to end among all dimensions. The individuals can be divided into three vertical facial patterns (hyperdivergent, hypodivergent, and normodivergent). Soft tissue growth correlates with underlying hard tissue growth [8]. Patients with long faces have weak and thin soft tissue morphology while cases with deep skeletal bites have strong and thick soft tissues [9,10].

A study on 195 lateral cephalograms reported that STC thickness values were most diminutive in hyperdivergent (7.47 ± 2.42 mm) and thickest in hypodivergent patients (11.78 ± 3.37 mm) [11]. The difference was statistically significant.

This study aims to measure and compare the thickness of the chin at different levels in various mandibular divergences. No previous data for our population can be found on soft tissue chin thickness measurements. This study will significantly help understand the soft tissue morphology of chin thickness and quantify visual treatment objectives in treatment planning.

## Materials and methods

This cross-sectional comparative study was conducted at the Department of Orthodontics, Sharif Medical & Dental College, Raiwind Road, Jati Umrah, Lahore, Pakistan from 1 January to 30 July 2022 on 195 cephalograms by non-probability consecutive sampling. A sample size of 195 cases was calculated with a 95% confidence interval and 6% margin of error. Ethical approval (Reference # 319-AD/PG/R/SMDC) was obtained from the Ethical Committee of Sharif Medical and Dental College. After an in-depth explanation of the study, informed consent was obtained from each participant.

Patients presenting for orthodontic treatment, of both genders, with ages ranging from 18 to 32 years, and Pakistani nationals were included. Patients with craniofacial deformity or syndrome, cleft lip and palate, previous orthodontic or orthognathic treatment, multiple missing teeth, and prostheses were excluded.

Age, gender, and cephalometric parameters were recorded from each participant. Standardized pre-treatment lateral cephalograms were taken with the “SoreDexCarnex-D Ceph” machine in a natural head position with lips relaxed. The lateral cephalogram of each subject was traced manually by a single operator in a standardized manner to avoid errors due to inter-operator variations. Standard 0.003 inch acetate tracing paper with a 0.3 mm mechanical pencil was used. The vertical facial divergence was calculated based on angular measurements of anterior cranial base length (SN) and mandibular plane (MP) as follows:

Horizontal growth pattern (hypodivergent): Low angle SN-MP < 27°

Normal growth pattern: Normal angle SN-MP (32° ± 5°)

Vertical growth pattern (Hyperdivergent): High angle > 37°

Soft tissue chin (STC) thickness was measured at three different levels:

Pog-Pog': Linear distance of bony pogonion (Pog) and its horizontal projection soft tissue pogonion (Pog')

Gn-Gn': Distance between bony gnathion (Gn) and its horizontal projection soft tissue gnathion (Gn')

**Figure 1:**
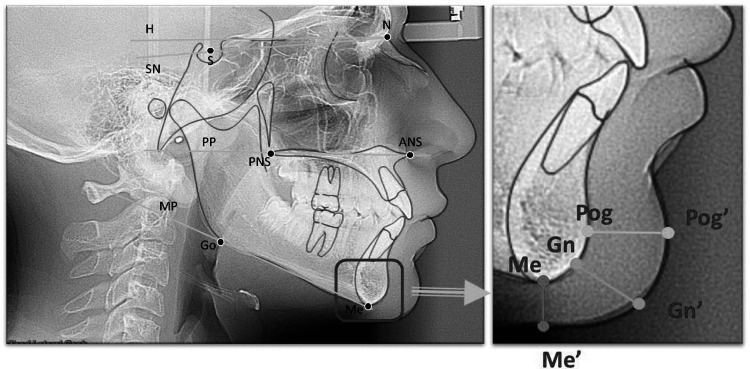
Radiographic anatomic landmarks of the mandible and chin used for measurement To the left: Mandibular plane (menton-gonion) and sella-nasion plane angle. To the right: Measurements of skin thickness at the chin were evaluated: hard tissue pogonion and the distance to its horizontal projection on the soft tissue (Pog-Pog’), distance from hard tissue gnathion to soft tissue gnathion (Gn-Gn’), and the distance from hard tissue menton to its vertical projection on the soft tissue (Me-Me’) Source: Macari AT, Hanna AE. Comparisons of soft tissue chin thickness in adult patients with various mandibular divergence patterns. The Angle Orthodontist. 2014. [11] (Open Access)

Me-Me': Distance between bony menton (Me) and soft tissue menton (Me') (Figure 1) [11]

Descriptive statistics were calculated for numerical data in terms of mean with SD and for categorical variables in terms of frequencies with percentages. STC parameters were compared among various facial vertical divergences (Normal, Low, High angle) using a one-way analysis of variance (ANOVA) test, and post hoc analysis was carried out using the Tukey test. Analysis was stratified among genders for controlling confounders. The level of significance was kept at p < 0.05.

## Results

The mean age was 21.66 ± 3.44 years, with the rest of the demographics shown in Table 1. The mean vertical angle was 32.09 ± 6.220. The mean distance from soft tissue (pog’) to hard tissue pogonion (Pog) was 11.14 ± 2.76 mm. The rest of the details are given in Table 2. The most common vertical facial pattern was normal (n=104, 53.33%), followed by high angle (n=50, 25.64%), and the least was low angle (n=41, 21.035).

**Table 1:** Distribution of gender and age group

	Characteristic	N (%)
Gender	Female	126 (64.62)
	Male	69 (35.38)
Age group	18 – 22 years	141 (72.3)
	23 – 27 years	33 (16.9)
	28 – 32 years	21 (10.8)

**Table 2:** Mean vertical angle and cephalometric soft tissue chin thickness Pogonion (Pog); Gnathion (Gn); Menton (Me); Sella Nasion to Mandibular Plane (SN-MP)

Characteristic	Mean ± SD
SN-MP angle (degree)	32.09 ± 6.22
Pog to pog’(mm)	11.14 ± 2.76
Gn to Gn’(mm)	7.75 ± 2.13
Me to Me’(mm)	7.19 ± 1.76

All three STC thickness distances significantly differed among vertical facial patterns (p<0.001). Multiple comparisons show that the distance between soft and hard tissue Pog was insignificant between low and normal angles (p=0.5). Similarly, no significant difference was observed for the distance between soft tissue and hard tissue menton between low and normal angles (p=0.4). The rest of the multiple comparisons were statistically significant (p<0.05) (Table 3).

**Table 3:** Comparison and post-hoc analysis of soft tissue chin thickness among various vertical facial patterns of all participants High: Hyperdivergent > 37°, Normal: Normal angle (32° ± 5°), Low: Hypodivergent < 27° Note: (*): Statistically significant (p-value<0.000) (a) One-way ANOVA (comparison of STC parameters among various facial vertical divergences (Normal, Low, High) (b)Post-hoc analysis: Difference between groups through the Tukey test (Difference of mean value of High vs. Low) ANOVA: analysis of variance

Variable (mm)	Mean ± SD			P-value^(^^a)^	Post-hoc analysis (P-value)^(^^b)^		
	High, N = 50	Low, N = 41	Normal, N = 104		High vs. Low	High vs. Normal	Low vs. Normal
Pog to pog’	9.40 ± 2.87	11.50 ± 2.26	11.83±2.53	<0.001^*^	<0.001	<0.001	0.5
Gn to Gn’	6.72 ± 1.26	8.72 ± 2.61	7.86 ± 2.06	<0.001^*^	<0.001	0.001	0.022
Me to Me’	6.28 ± 1.25	7.32 ± 1.71	7.58 ± 1.85	<0.001^*^	0.004	<0.001	0.4

In males, similar results were found in the overall study, except that no statistical difference was found for soft tissue chin thickness parameters low versus normal angle (p>0.05) (Table 4). Among females, the difference was statistically significant except for Gn-Gn’ between high and normal angle (p=0.13), Pog-Pog’ between low and normal angle (p=0.7), and Me-Me’ between low and normal angle (p=0.5) (Table 5).

**Table 4:** Comparison and post-hoc analysis of soft tissue chin thickness among various vertical facial patterns in males High: Hyperdivergent > 37°, Normal: Normal angle (32° ± 5°), Low: Hyperdivergent < 27° Note: (*): Statistically significant (p-value<0.000) (a) One-way ANOVA (comparison of STC parameters among various facial vertical divergences (Normal, Low, High angle) (b) Post-hoc analysis: Difference between groups through the Tukey test ANOVA: analysis of variance

				P-Value^(a)^	Post-hoc analysis^(b)^ (Difference of mean values between high, low, and normal)		
Variable (mm)	High, N = 16	Low, N = 11	Normal, N = 42		High vs. Low	High vs. Normal	Low vs. Normal
Pog to pog’	9.09 ± 2.44	11.82 ± 2.48	12.18 ± 2.52	<0.001*	0.007	<0.001	0.7
Gn to Gn’	6.12 ± 0.79	8.8 ± 2.27	8.14 ± 2.25	<0.001*	0.001	0.001	0.3
Me to Me’	6.25 ± 1.05	7.73 ± 2.10	7.76 ± 1.74	<0.001*	0.027	0.003	>0.9

**Table 5:** Comparison and post-hoc analysis of soft tissue chin thickness among various vertical facial patterns in females High: Hyperdivergent > 37°, Normal: Normal angle (32° ± 5°), Low: Hyperdivergent < 27° Note: (*): Statistically significant (p-value<0.000) (a) One-way ANOVA (comparison of STC parameters among various facial vertical divergences (Normal, Low, High angle) (b) Post-hoc analysis: Difference between groups through the Tukey test ANOVA: analysis of variance

					Post-hoc analysis^(b)^ (Difference of mean values between high, low, and normal)		
Variable (mm)	High, N = 34	Low, N = 30	Normal, N = 62	P-value^(a)^	High vs. Low	High vs. Normal	Low vs. Normal
Pog-Pog’	9.54 ± 3.07	11.38 ± 2.20	11.60 ± 2.53	<0.001*	0.006	<0.001	0.7
Gn-Gn’	7.00 ± 1.35	8.68 ± 2.76	7.66 ± 1.92	<0.001*	0.001	0.13	0.025
Me-Me’	6.29 ± 1.34	7.17 ± 1.56	7.45 ± 1.93	<0.001*	0.043	0.002	0.5

## Discussion

Evaluation of soft tissues is crucial in diagnosing conditions, planning treatments, and achieving facial harmony. Research has shown that considering soft tissue features significantly enhances treatment outcomes. The soft tissue profile is extensively studied in orthodontics, often using lateral cephalometric radiographs. It's commonly assumed that the shape of the soft tissue outline greatly influences facial aesthetics. Studies have indicated that not only the lips but also the position of the chin has a significant effect on the aesthetics of the lower face [12].

This cross-sectional study was carried out to compare STC thickness in various mandibular divergence patterns. Our findings showed statistical differences in STC thickness parameters among various vertical facial divergences. The vertical growth in patients can affect their treatment planning. This sort of growth stays longer than sagittal and transverse growth. In orthodontics, three vertical facial patterns can be seen: normo-divergent, hypo-divergent, and hyper-divergent. Growth in the vertical dimension can affect the soft tissues of the face [13]. Chin morphology can have a significant impact on facial profile and a retrusive chin can render the profile convex.

There is a scarcity of literature regarding soft tissue cephalometric norms specifically for individuals of Pakistani descent. Existing studies in this area have primarily concentrated on dentofacial patterns rather than conducting comprehensive soft tissue analyses. One such study by Sattar et al. (2018) compared Pakistani adults with established standards in orthodontics [14]. Their findings indicated that the Pakistani population tends to exhibit a more convex soft tissue profile in comparison to the established standards of Tweed and Steiner. This observation was attributed in part to variations in the shape of the nose and the morphology of the lips among Pakistani individuals. However, despite these initial findings, further research is needed to fully elucidate the soft tissue cephalometric norms specific to the Pakistani population.

Our study found that the most common vertical facial pattern was normal (n=104, 53.33%), followed by high angle (n=50, 25.64%), and the least was low angle (n=41, 21.035). The mean STC thickness in the high-angle group was 7.940 ± 2.87 mm, 11.50 ± 2.26 mm in the low-angle group, and 11.83 ± 2.53 mm in the normal group. Macari et al. (2014) conducted a study on 190 patients to compare STC in various vertical facial patterns. All STC thicknesses had the highest measurements in the hypodivergent group and gradually decreased across the groups, the lowest being in the hyperdivergent group. These results are consistent with our findings [11]. Another study by Ashraf K et al. (2018) concluded that soft tissue chin thickness (Pog-Pog'), (Gn-Gn'), and (Me-Me') was found to be statistically significant only between the hyperdivergent and hypodivergent groups [1].

Our study aligns with the findings of Celikoglu et al. (2014) who concluded that in both women and men, the high-angle group exhibited the lowest soft tissue thickness values. Specifically for women, the thickness values at pogonion were significantly smaller in the high-angle group. Furthermore, the low-angle and normal-angle groups demonstrated comparable thickness values [9]. Somaiah et al. (2017) observed statistically significant differences between men and women across different facial types. In the low mandibular divergence pattern, a highly significant difference was observed for Pog-Pog' between genders. In the medium-low divergence pattern, there were no significant differences in Pog-Pog', Gn-Gn', and Me-Me'. However, in the medium-high divergence pattern, a highly significant difference was observed for Pog-Pog' between genders, and the mean for Gn-Gn' was significant. In the high mandibular divergence pattern, there were no significant differences in Pog-Pog', Gn-Gn', and Me-Me' between genders [15].

Comparing our results with those of other researchers is challenging due to the scarce literature available on this topic. The variances between our findings and those of other studies could stem from differences in race, age groups studied, and the sizes of the samples. There's a notable gap in studies that examine and compare the thickness of the soft tissue around the chin and the length and thickness of the upper lip across different vertical discrepancies. Additional research in this area could offer valuable insights, enhancing our ability to diagnose and develop treatment plans for orthodontic patients more effectively.

The limitations of our study are primarily related to the sample size and the distribution of participants. First, the limited sample size may restrict the generalizability of our findings to the broader population. Second, there was an unequal distribution of gender within our study sample, which could potentially introduce bias or limit the applicability of our results across genders. Lastly, the unequal distribution of facial types among participants might have influenced the outcomes, possibly affecting the reliability of comparisons across different facial morphology categories. These limitations highlight the need for caution in interpreting our results and underscore the importance of conducting future research with larger, more evenly distributed samples to validate and expand upon our findings.

## Conclusions

Within the limitations of this study, the thickness of the soft tissue chin has a significant association with vertical facial divergence. While planning orthognathic surgery of the mandible or genioplasty, due consideration should be given to vertical divergence of the face to avoid unwanted facial changes.
